# Streptococcus pyogenes: An Unusual Cause of Urethritis

**DOI:** 10.7759/cureus.69267

**Published:** 2024-09-12

**Authors:** Francisca Bartilotti Matos, Mafalda Ribeirinha, Rui Salvador, Joana Fragoso, Luís Malheiro

**Affiliations:** 1 Infectious Diseases Department, Unidade Local de Saúde Gaia e Espinho, Vila Nova de Gaia, PRT; 2 Clinical Pathology Department, Unidade Local de Saúde Gaia e Espinho, Vila Nova de Gaia, PRT; 3 Internal Medicine Department, Unidade Local de Saúde Gaia e Espinho, Vila Nova de Gaia, PRT; 4 Infectious Diseases Department, Unidade Local de Saúde Gaia e Espinho, Vila Nova De Gaia, PRT

**Keywords:** herpes simplex virus, non-gonococcal urethritis, sexually transmitted infections, streptococcus pyogenes, urethritis

## Abstract

Nongonococcal urethritis is a very common infection in men. Though *Streptococcus pyogenes* has frequently been associated with other genital tract infections, it has very rarely been reported as a causative agent of urethritis.

We report a case of a healthy 31-year-old man who presented to the emergency department with symptoms of urethritis and penile ulcers. Culture of both urine and urethral exudate were positive for *S. pyogenes* sensitive to penicillin. A swab of the ulcer was positive for herpes simplex virus 2. He was treated with a seven-day course of amoxicillin and acyclovir, with full resolution of symptoms.

To the best of our knowledge, this is the first case report of *S. pyogenes* urethritis transmitted through sex between men. This organism is a possible and probably underreported agent of urethritis. This case underscores the importance of microbiological testing in the treatment of urethritis.

## Introduction

Nongonococcal urethritis (NGU) is one of the most common sexually transmitted infections in men [1]. Most NGU cases are caused by *Chlamydia trachomatis* and *Mycoplasma genitalium*, but in up to half of the cases, an etiology is not identified [2].

*Streptococcus pyogenes* is a gram-positive bacterial pathogen responsible for a broad range of disease manifestations, being a common cause of tonsillitis and skin and soft tissue infections, but also otitis media, endocarditis, and osteomyelitis [3]. Concerning the genital tract, it has been associated with vulvovaginitis, mainly in prepubescent women [4], and with balanitis and balanoposthitis in men, also mostly in children [5]. In adults, *S. pyogenes* balanoposthitis has been associated with orogenital sexual practices [6].

There are, however, very few reports of urethritis caused by this agent. Previous isolated case reports include one in Spain in 1998 [7], and one in France in 2006 [8]. They were both referents to men under the age of 40 years, one assumed to have been transmitted through heterosexual practices, the other through contact with a case of impetigo.

We present a confirmed case of *S. pyogenes* urethritis (isolated in urine and urethral exudate) in a symptomatic man, following unprotected sexual intercourse, associated with a herpes simplex virus 2 (HSV-2) infection.

## Case presentation

A 31-year-old man with no past medical history presented to the emergency department with a one-week history of purulent urethral exudate associated with several small painful genital ulcers. He denied testicular or perineal pain. Regarding his recent sexual history, he mentioned having unprotected sexual intercourse with a new male sexual partner two weeks prior. On physical examination, he was afebrile with normal vital signs. There were two white-bottomed small painful carbuncles (3 mm) on the base of the glans of his penis and one on the internal leaflet of the foreskin. A purulent urethral discharge was also described.

At the emergency department, initial blood tests showed a normal full blood count with a mildly raised C-reactive protein (3.5 mg/dL, normal < 0.5 mg/dL), urinary microscopy with 34 leucocytes/field, negative antigen/antibody HIV test, and negative syphilis screening (electrochemiluminescence immunoassay analyzer). With these preliminary results, he was medicated empirically with acyclovir, ceftriaxone 500 mg (single dose, intramuscular injection), and doxycycline (100 mg, orally, twice a day, for seven days) and discharged for follow-up in the infectious diseases outpatient clinic. Other pending tests requested at the emergency department included urinary nucleic acid amplification testing (NAAT) for *Chlamydia trachomatis* and *Neisseria gonorrhoeae*, urine and urethral exudate culture, and an Allplex genital ulcer real-time polymerase chain reaction (PCR) multiplex assay (Seegene Inc, Seoul, Republic of Korea) of a swab of the base of the ulcer for common sexually transmitted agents.

**Figure 1:**
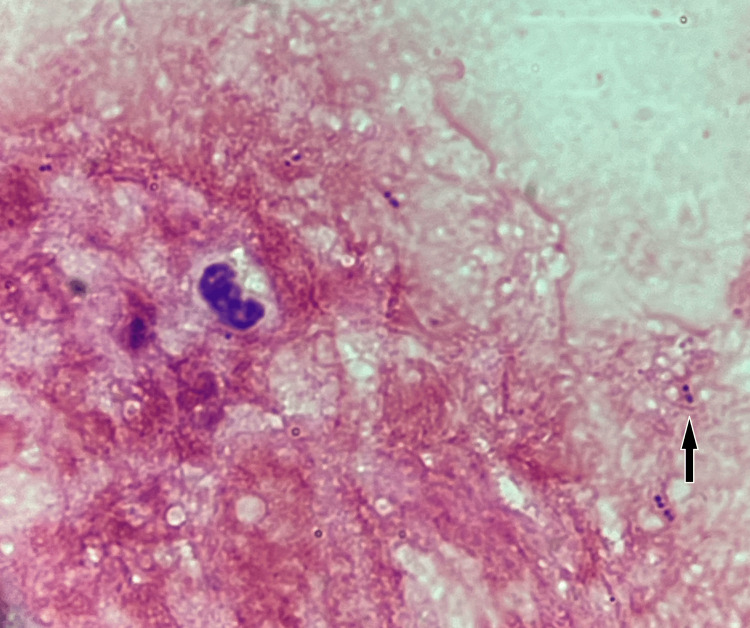
Microscopic examination of a Gram-stained smear of the urethral exudate revealed small Gram-positive cocci (arrow).

These results were available the week after presentation and showed a negative urine NAAT for *C. trachomatis* and *N. gonorrhoeae*. Gram stain of the urethral exudate revealed Gram-positive cocci-shaped bacteria in pair and chain arrangement (Figure 1).

**Figure 2:**
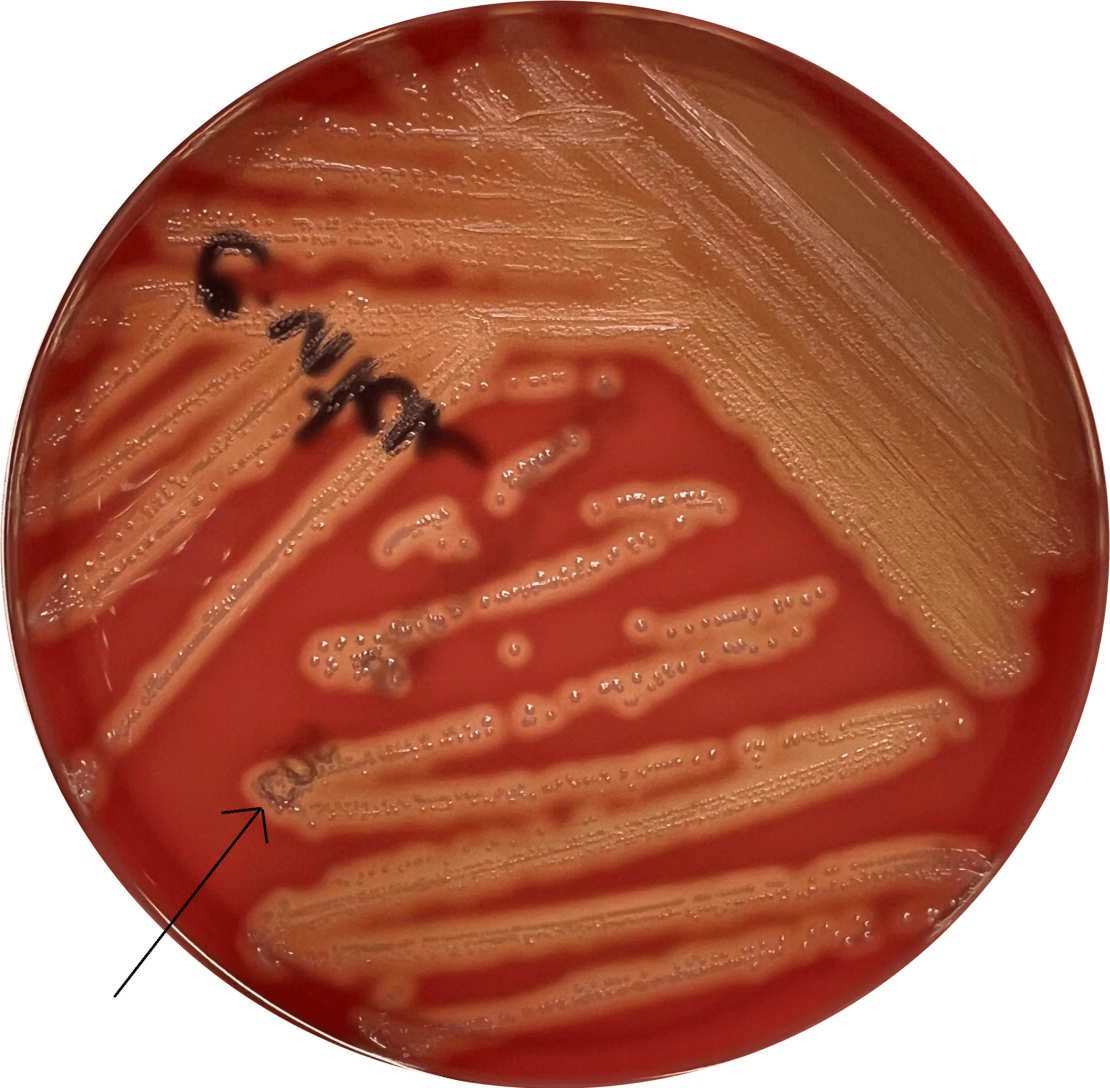
Culture of the urethral exudate after 24 hours of incubation showing the growth of colonies of Streptococcus pyogenes (arrow) on Columbia agar with 5% sheep blood.

The urethral exudate culture was incubated using Columbia agar with 5% sheep blood (bioMérieux SA, Marcy-l’Etoile, France) at 35ºC in 5% CO2 and urine culture was incubated using Columbia CNA agar with 5% sheep blood (bioMérieux SA, Marcy-l’Etoile, France) at 37ºC. After 24 hours of incubation, small grey β-hemolytic round-shaped colonies were visible on both cultures, which were catalase and coagulase-negative (Figure 2). Both isolates were subjected to automated mass spectrometry microbial identification using the VITEK® MS (bioMérieux, Inc., Durham, NC) and were identified as *Streptococcus pyogenes*. Antibiotic susceptibility testing was conducted in both isolates using the VITEK®2 Streptococci Card. Both urine urethral exudate culture isolates were sensitive to penicillin, erythromycin, and clindamycin (Table 1). PCR testing of the basis of the ulcer was positive for HSV-2.

**Table 1:** Antibiotic susceptibility testing of the Streptococcus pyogenes isolate. Antibiotic sensitivity, minimum inhibitory concentrations (MIC), and cutoffs for Streptococcus Group A using the VITEK® 2 Streptococci Card. The European Committee on Antimicrobial Susceptibility Testing (EUCAST) cutoffs were used to categorize into two categories: susceptible with standard dosing regimen (S) or resistant (R). Breakpoint tables for interpretation of MICs and zone diameters. Version 14.0, 2024. http://www.eucast.org.

Streptococcus Group A				
Antibiotics	EUCAST		VITEK® 2	
	S≤	R>	MIC	Sensitivity
Penicillin	0.25	0.25	≤0.06	S
Erythromycin	0.25	0.25	≤0.12	S
Clindamycin	0.5	0.5	≤0.25	S

Based on his symptoms and test results, he was diagnosed with *S. pyogenes* urethritis and with HSV-2 infection and medicated with amoxicillin for seven days, completing also a seven-day course of acyclovir that had been commenced at the emergency department. He had full resolution of symptoms. On his wish, and after confirming he fulfilled the referral criteria, he was also referred to start the pre-exposure prophylaxis clinic.

## Discussion

*S. pyogenes* is an exceedingly rare cause of urethritis, and, to the best of our knowledge, this is the first case report of *S. pyogenes* urethritis assumed to have been transmitted through sex between men. This case, besides showing an unusual presentation and transmission of a common pathogen, solidifies the importance of microbiology testing in urethritis.

Over the past years, we have been witnessing a change in the epidemiology of sexually transmitted infections. Considering the increasing amount of resistance in common urethritis agents, more pronounced in *N. gonorrhoeae* [9], it is fundamental to collect and follow up on microbiological results to treat this kind of infection. If possible, treatment should be directed to a pathogen and not presumptive.

*S. pyogenes* has been found to dominate the urethral microbiota of men with urethritis [10] and it has been proposed as a causative agent for sexually transmitted balanoposthitis [5]. Studies comparing idiopathic urethritis with controls showed a high prevalence of *S. pyogenes* genome when urethral microbiota was evaluated using 16S rRNA gene sequencing [11].

Though there are very few reports of *S. pyogenes* infection, it is possible that this infection goes frequently undiagnosed, as many centers that manage urethral infections do not have access to a microbiology laboratory and treat them empirically. Even in hospitals with laboratory capacity, it is hard to identify uncommon bacteria in urine, as it is a polymicrobial sample and it takes experienced microbiologists to distinguish disease-causing pathogens from colonizing agents. A possible fast screening tool to diagnose *S. pyogenes* infection is a rapid antigen test in patients presenting with urethritis [12]. This is important, as the preferred treatment for these infections is different from the common antibiotic combination used to presumptively treat urethritis. Though ceftriaxone and azithromycin are antibiotics that would also cure an *S. pyogenes* infection, their larger spectrum of action, when compared to penicillin or amoxicillin, makes them a less ideal option.

Potential infectious routes for *S. pyogenes* urethritis include orogenital sexual intercourse, autoinoculation from asymptomatic oropharyngeal carriers, asymptomatic anal carriage, and extension of anal dermatitis [10]. Though we cannot confirm that this has been a sexually transmitted case, the association with infection by herpes simplex 2 and the epidemiological context make this the most likely hypothesis. Unfortunately, it was not possible to test the partner’s oropharynx to confirm our hypotheses.

Similar to previously reported cases of *S. pyogenes* urethritis, oral amoxicillin was an effective treatment for *S. pyogenes* urethritis.

## Conclusions

This is a rare case of sexually transmitted *S. pyogenes* urethritis, associated with HSV-2 infection. With the challenges posed by antimicrobial resistance and harder-to-treat urethritis, it is more and more important to be aware of possible unusual pathogens to direct and optimize treatment. In patients who have a history of orogenital sexual intercourse, it is fundamental to have common throat bacteria in mind when treating these infections.
